# Right coronary sinus of Valsalva pseudoaneurysm after a motorcycle accident: A case report

**DOI:** 10.1097/MD.0000000000041341

**Published:** 2025-01-24

**Authors:** Yong Cheng, Yu Zhang, Zixuan Liu, Ziwei Wang, Tao Shuai

**Affiliations:** a Department of Radiology, West China Hospital, Sichuan University, Chengdu, Sichuan, China.

**Keywords:** case report, pseudoaneurysm, sinus of Valsalva, trauma

## Abstract

**Rationale:**

Traumatic pseudoaneurysm of the sinus of Valsalva (PSV) is a rare but life-threatening condition, often resulting from blunt chest trauma. Rapid progress and a high risk of rupture highlight the importance of prompt diagnosis and intervention. We present a case of a rare pseudoaneurysm linked to the right coronary sinus after blunt chest trauma.

**Patient concerns:**

A 54-year-old male presented with chest pain, loss of consciousness, and coma following a motorcycle accident. Initial management included invasive ventilation at a local hospital before transfer for specialized care.

**Diagnoses:**

Computed tomography angiography and transthoracic echocardiography confirmed a blunt traumatic PSV, along with an 8th rib fracture, findings later corroborated during open thoracotomy.

**Interventions:**

The pseudoaneurysm was surgically treated with an open thoracotomy, and the defect was sealed using a pericardial patch.

**Outcomes:**

The patient experienced an uneventful recovery, with follow-up confirming the restoration of normal daily activities.

**Lessons:**

Traumatic PSV, though rare, demands heightened clinical awareness in cases of blunt chest trauma. Noninvasive computed tomography angiography serves as a rapid and reliable diagnostic tool. Early surgical intervention remains the definitive treatment to prevent catastrophic outcomes.

## 1. Introduction

Pseudoaneurysms of the sinus of Valsalva (PSV) are extremely rare, with both congenital and acquired causes. Acquired PSV is mainly due to infection, trauma, and iatrogenic injury.^[1]^ PSV can occur in the right, left, or noncoronary sinus. Previous literature shows that nontraumatic pseudoaneurysms most often affect the right coronary sinus, followed by the noncoronary sinus, with the left coronary sinus being the least involved.^[2–4]^ However, traumatic pseudoaneurysms are most commonly found in the left coronary sinus, with the right coronary sinus being the least affected.^[2]^ We present a case of a pseudoaneurysm in the right coronary sinus of Valsalva following chest trauma.

## 2. Case presentation

A 54-year-old man presented with chest pain 16 hours after a motorcycle accident, along with episodes of unconsciousness and coma. Initial care at a local hospital included endotracheal intubation and invasive ventilator-assisted ventilation. Enhanced chest computed tomography imaging revealed a ruptured ascending aorta with a pseudoaneurysm. Due to the patient’s worsening condition, he was promptly transferred to our facility for urgent treatment.

**Figure 1.**
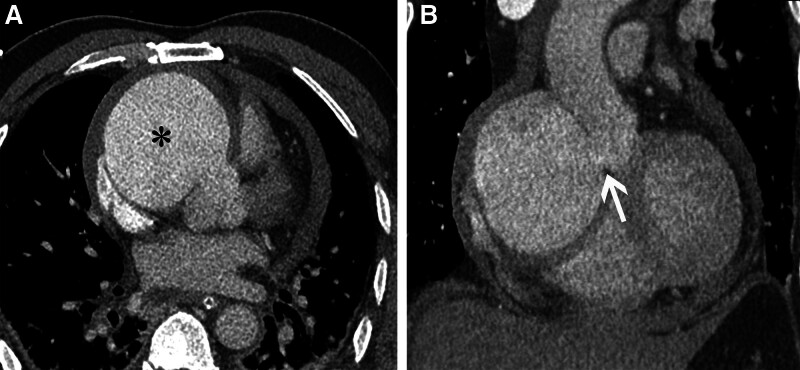
Preoperative chest CTA images of the patient. (A) The axial section shows a spherical high-density contrast-filled image (black asterisk). (B) Oblique coronal section revealing a rupture measuring 1.6 × 2.0 cm at the right coronary sinus (white arrow). CTA = computed tomography angiography.

Upon admission to our hospital, thorough laboratory tests showed: B-type natriuretic peptide precursor at 481 ng/L, procalcitonin at 0.06 ng/mL, myoglobin at 202.80 ng/mL, creatine kinase isoenzyme MB at 17.03 ng/mL, troponin-T at 760.6 ng/L, and hemoglobin at 127 g/L. An emergent chest computed tomography angiography (CTA) revealed moderate pericardial effusion and a large spherical pseudoaneurysm measuring 7.5 × 7.0 × 6.3 cm in the right atrioventricular groove (Fig. 1). Additionally, a rupture measuring 1.6 × 2.0 cm was identified in the right coronary sinus of the ascending aorta (Figs. 1B and 3B). The middle segments of the right coronary artery were not visualized, whereas the proximal and distal segments were clearly delineated. Transthoracic echocardiography identified a tumor-like anechoic area, measuring 7.5 × 7.0 × 6.6 cm at the sinotubular junction, with evidence of a shunt from the aorta to the pseudoaneurysm, exhibiting a peak flow velocity of 1.1 m/s.

**Figure 2.**
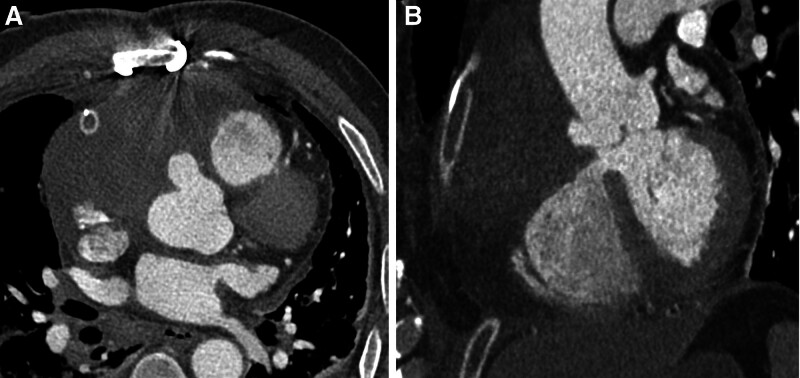
Postoperative chest CTA images of the patient. Postoperatively, the pseudoaneurysm’s rupture site was successfully sealed with a pericardial patch. CTA = computed tomography angiography.

**Figure 3.**
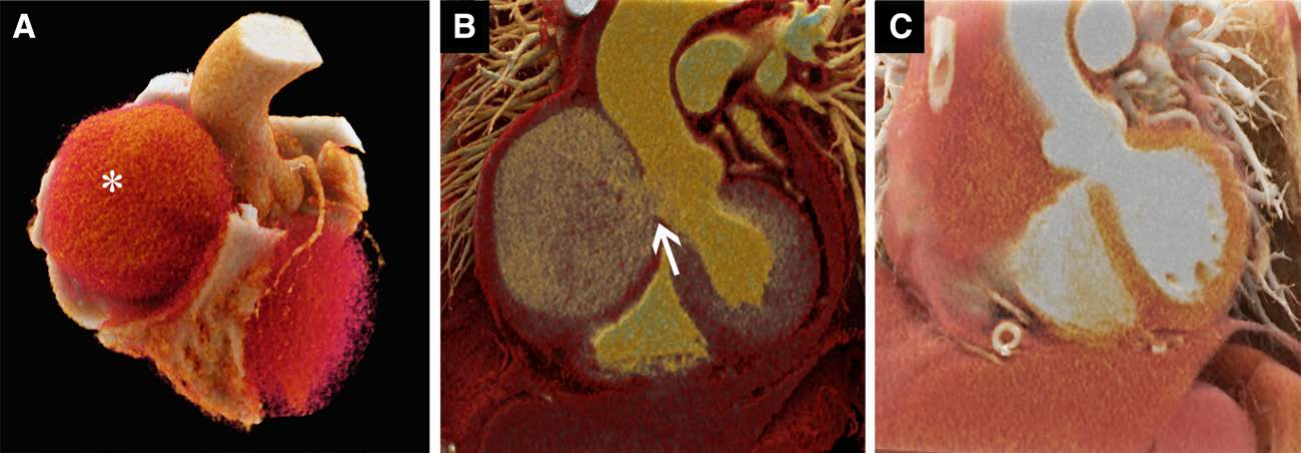
Cinematic renderings based on CTA. (A) Showing a pseudoaneurysm located in the right atrioventricular groove area (white asterisk) and (B) displaying a rupture of 1.6 × 2.0 cm in the right coronary sinus of the ascending aorta (white arrow). After surgery, (C) shows that the pseudoaneurysm channel had closed. CTA = computed tomography angiography.

There was no prior history of trauma or chest issues in the patient’s medical records, nor had he undergone any relevant medical examinations. Based on the ultrasound and CTA findings, the patient was diagnosed with a pseudoaneurysm of the right coronary sinus of Valsalva due to blunt trauma. Surgical options, including Bentall surgery and coronary artery bypass grafting, were discussed.

The patient underwent thoracotomy and exploration, which revealed a 12.0 × 10.0 cm pseudoaneurysm. Around 100 mL of acute pericardial hematoma and a small thrombus were found within the pericardium. The right coronary artery’s normal origin was seen above the rupture site. A 2.5 × 2.0 cm pericardial patch was used to close the tear in the right coronary sinus of Valsalva, avoiding the need for coronary artery resection or bypass surgery. The patient remained in a coma following the operation, with stable vital signs, and was transferred to the intensive care unit for invasive ventilator-assisted ventilation, where he received symptomatic and supportive treatment. Postoperative transthoracic echocardiography showed that the pseudoaneurysm had resolved, with no abnormal blood shunting observed at the aortic root. One day after surgery, the patient regained consciousness, and a follow-up coronary CTA confirmed the disappearance of the pseudoaneurysm. The CTA also demonstrated excellent visualization of the left anterior descending and left circumflex coronary arteries, although the proximal segment of the right coronary artery was not visible, while the distal segment was well visualized (Fig. 2). Eight days after surgery, the patient was transferred to another hospital for rehabilitation, and the details of his treatment at that facility remain unknown. At the most recent follow-up, the patient was able to resume normal daily activities.

## 3. Discussion

This article reports a rare case of a pseudoaneurysm of the right coronary artery sinus of Valsalva caused by closed thoracic trauma due to a motorcycle accident. The diagnosis was confirmed by CTA and transthoracic echocardiography, and further validated by exploratory thoracotomy. The patient was successfully treated by sealing the rupture with a pericardial patch. Six-month follow-up showed excellent recovery.

The PSV are exceedingly rare, with causes broadly divided into congenital and acquired factors. Acquired causes mainly included infection (endocarditis, syphilis, and tuberculosis), trauma, and iatrogenic injury (cardiac surgery and vascular interventions).^[1,2,5–7]^ In this case, the patient had no history of trauma, surgical, or infections prior to the accident, making it likely that the pseudoaneurysm resulted from closed thoracic trauma.

The incidence of aortic injury caused by blunt thoracic trauma is very low, with some literature reporting it as low as 0.022%. Pseudoaneurysms caused by thoracic trauma are even rarer, with few studies analyzing the mechanisms of their formation. It is possible that patients with thoracic trauma may deteriorate quickly and die before treatment, thus underestimating the true incidence of traumatic pseudoaneurysms of the aortic sinus. Aortic injury can occur due to mechanisms like chest compression or high-acceleration impacts, where tensile stress leads to vascular damage. Traumatic pseudoaneurysms are thought to occur when forces fall below the threshold for vessel rupture, causing intimal injury while the adventitia remains intact.^[8–11]^ The mechanism behind pseudoaneurysm formation in the sinus of Valsalva likely involves increased pressure in the aortic root during diastole following blunt chest trauma, leading to tears in the Valsalva sinus.^[2]^ However, the specific mechanism remains unclear, and replicating relevant experiments is challenging.

The most dangerous complication of acute traumatic PSV is rupture, which can lead to immediate death. Unruptured PSV can present with chest pain or complete heart block, mainly due to compression from the pseudoaneurysm or the effect of blood and inflammatory cells on the conduction system. Chronic aortic injury after thoracic trauma may also occur and be diagnosed up to 18 years later. While such patients may be asymptomatic, the formation of a pseudoaneurysm increases the risk of rupture.^[12,13]^

Diagnosing traumatic pseudoaneurysms relies on imaging techniques like ultrasonography, computed tomography, and cardiac magnetic resonance. Ultrasound typically shows the pseudoaneurysm as an echo-free mass, and blood flow imaging helps differentiate it from other pathologies. While cardiac magnetic resonance is less commonly used in urgent cases due to its longer scanning time and less availability in emergency settings, CTA is the preferred diagnostic tool. CTA offers detailed localization, sizing, and anatomical relationships of pseudoaneurysms with surrounding structures. Additionally, three-dimensional reconstructions via cinematic rendering techniques enhance visual comprehension, aiding clinicians in planning treatment and communication with patients and their families (Fig. 3).

Traumatic pseudoaneurysms progress rapidly and carry high mortality, particularly especially if ruptured. Surgical is the primary treatment. In this case, the patient underwent successful surgery and recovered well. However, there are several limitations to this report. First, our hospital was not the initial medical facility to receive the patient, and the exact circumstances at the site of the motorcycle accident remain unclear, which limits a comprehensive understanding of the trauma. Like most previous studies, we were unable to analyze the underlying mechanism behind the formation of the pseudoaneurysm. Additionally, the follow-up period for this patient was relatively short, which may not provide sufficient insight into long-term outcomes and potential complications. Issues may emerge over a longer period that are not addressed in this case report. Finally, the treatment approach was based on the specific conditions and resources available at our institution and may not be applicable to all clinical settings. Despite this, a review of the current literature did not reveal a clearly superior treatment strategy for traumatic PSV.

In conclusion, the case we report provides valuable experience for the diagnosis and treatment of similar patients. This case emphasizes the importance of comprehensive clinical vigilance and detailed initial imaging of the thoracic aorta in patients with recent blunt chest trauma. Early detection and intervention are critical to prevent life-threatening complications from traumatic pseudoaneurysms of the sinus of Valsalva.

## 4. Conclusion

Although traumatic PSV is relatively rare, clinicians should maintain a high index of suspicion for this condition in patients with closed thoracic trauma. noninvasive imaging is critical in diagnosing PSV, evaluating its severity, and defining its relationship with surrounding anatomical structures. Surgical intervention remains the gold standard for treating PSV, and timely management can lead to favorable outcomes.

## Acknowledgments

The authors offer acknowledgments to Xinyue Chen from Siemens Healthineers, for her careful English editing and proofreading of this manuscript.

## Author contributions

**Conceptualization:** Tao Shuai.

**Formal analysis:** Yong Cheng, Zixuan Liu, Ziwei Wang.

**Writing – original draft:** Yong Cheng.

**Writing – review & editing:** Yu Zhang.
